# The pesticide flupyradifurone impairs olfactory learning in Asian honey bees (*Apis cerana*) exposed as larvae or as adults

**DOI:** 10.1038/s41598-017-18060-z

**Published:** 2017-12-19

**Authors:** Ken Tan, Cao Wang, Shihao Dong, Xinyu Li, James C. Nieh

**Affiliations:** 1 Key Laboratory of Tropical Forest Ecology, Xishuangbanna Tropical Botanical Garden, Chinese Academy of Science, Kunming, Yunnan Province 650223 China; 2 Eastern Bee Research Institute, Yunnan Agricultural University, Heilongtan, Kunming, Yunnan Province 650223 China; 3 Division of Biological Sciences Section of Ecology, Behavior, and Evolution, University of California, San Diego, La Jolla, California, USA

## Abstract

Relatively little attention has focused on how pesticides may affect Asian honey bees, which provide vital crop pollination services and are key native pollinators. We therefore studied the effects of a relatively new pesticide, flupyradifurone (FLU), which has been developed, in part, because it appears safer for honey bees than neonicotinoids. We tested the effects of FLU on *Apis cerana* olfactory learning in larvae (lower dose of 0.033 µg/larvae/day over 6 days) and, in a separate experiment, adults (lower dose of 0.066 µg/adult bee/day) at sublethal, field-realistic doses given over 3 days. A worst-case field-realistic dose is 0.44 µg/bee/day. Learning was tested in adult bees. The lower larval dose did not increase mortality, but the lower adult dose resulted in 20% mortality. The lower FLU doses decreased average olfactory learning by 74% (larval treatment) and 48% (adult treatment) and reduced average memory by 48% (larval treatment) and 22% (adult treatment) as compared to controls. FLU at higher doses resulted in similar learning impairments. The effects of FLU, a pesticide that is reported to be safer than neonicotinoids for honey bees, thus deserve greater attention.

## Introduction

Global concern has grown over the effects of pesticides^1^, particularly neonicotinoids, on beneficial pollinators^2^. Honey bees play an important role in pollinating crops^3^ and are therefore widely and common exposed to pesticides, which have multiple individual effects, even at sublethal doses^4–6^ and can interact with other factors that reduce bee health, including parasite infections and viral diseases^7^. Interest has therefore grown in new pesticides that could reduce this harm. However, researchers have largely focused upon a single species, *Apis mellifera* ^8^, even though Asian honey bee species are also vital. *Apis cerana* has a wide range extending from China to India, including southern and eastern Asia^9^ over which it provides important ecosystem services^3,10,11^ and is a key pollinator of native plant species^10,12^. Although *A*. *cerana* is evidently more resistant to some parasites^13^ and pathogens^14^ than *A*. *mellifera*, the health of this species is also of concern^15–17^. More than two million managed colonies of *A*. *cerana* are used in China for crop pollination and honey production^10^. *Apis cerana* is thus exposed to multiple pesticides^18^, including newly registered compounds.

Flupyradifurone (FLU) is a butenolide systemic insecticide whose chemical structure was designed based upon a natural compound, stemofoline, found in the Asian medicinal plant, *Stemo japonica* ^19^. Like the neonicotinoids, stemofoline acts upon insect nicotinic acetylcholine receptors (nAChRs)^20^. Modifications of stemofoline’s structure resulted in the creation of FLU^19^, which was first commercially registered in Guatemala and Honduras in 2014^19^. Since then, FLU has been registered for use on a wide variety of crops in the USA^21^, Europe^22^, and China^23^. Common agricultural applications include use on citrus, cocoa, cotton, grapes, hops, pome fruits, potatoes, soybeans, and ornamental plants^19^.

FLU has a far lower binding affinity to insect nAChRs than neonicotinoids, but can be effective against plant sucking insects, particularly those that have developed resistance to neonicotinoids^19^. In addition, studies suggest that FLU has a relatively low impact on *A*. *mellifera* ^24^. For example, *A*. *mellifera* colonies placed in plots adjacent to fields sprayed with FLU were exposed to low levels of FLU, but did not have different colony strength parameters (numbers of adult workers, eggs, brood cells, food storage cells, and colony mass) as compared with controls^25^. However, to date, no studies have examined the impact of FLU on *A*. *cerana*, even though a meta-analysis suggested that *A*. *cerana* is more susceptible to some pesticides than *A*. *mellifera* ^26^. Neonicotinoid pesticides, which also act upon nAChRs, may exert a stronger detrimental effect^27^, up to 10-fold higher^28^, on *A*. *cerana* as compared to *A*. *mellifera*. However, the meta-analysis conducted by Arena and Sgolastra^26^ found that neonicotinoids had a similar effect upon both species. In general, the effects of nAChR agonists on different target species are difficult to predict *a priori* ^29^, and experiments are therefore needed.

The majority of pesticide studies conducted with honey bees focus on lethality and overall colony effects^30^. However, the effects of pesticides should be assessed on multiple aspects of pollinator health and biology^31^. For example, honey bees are noted for their olfactory learning^32^. Olfactory learning is key to colony food intake and the pollination services provided by honey bees because it allows bees to associate odors with nectar and pollen rewards, thereby helping them to find rewarding food and assisting pollination by facilitating floral constancy^32^. Multiple studies have shown that neonicotinoids and other pesticides can impair olfactory learning in bumble bees^33^ (though not all ref.^34^) and in honey bees (*A*. *mellifera* ^35–42^ and *A*. *cerana* ^43,44^). To date, no published studies have examined the effects of FLU on honey bee learning.

Honey bees can be exposed to pesticides at multiple life stages, including the larval stage^45^, which may be more sensitive to such toxins^37,43^. Yang *et al*.^37^ demonstrated that *A*. *mellifera* fed imidacloprid as larvae had reduced olfactory learning when they became adults, even though the larvae were fed much smaller imidacloprid doses than are required to impair adult learning^36,37,46^. It was unknown if FLU would similarly affect *A*. *cerana* larvae or adults. We thus tested the sublethal effects of FLU on survival and olfactory learning when bees were exposed as larvae or as adults.

## Results

FLU residues are detectable from the inflorescences of a wide variety of crops (including apple) for 7 days after foliar application^24^. A high field-realistic dose of FLU is 0.44 µg/forager/day (from bees foraging on apple blossoms)^24^. All of our lower FLU doses and exposure durations were field-realistic, based upon this forager data (0.033 µg/larvae/day and 0.066 µg/adult bee/day). Full details on doses and concentrations are shown in Table 1.

**Table 1:** Doses and concentrations of flupyradifurone (FLU) fed to larvae and adults. Field-realistic doses are 0.44 µg/bee/day^24^ and concentrations of 4.1–4.3 ppm in forager honey stomachs MRID 48844516 and MRID 48844517 in ^24^. Calculations of ppm are based upon the density of pure sucrose solution (1.127 g/ml) at 1 ATM and 21 °C. The molecular weight of FLU is 288.68 g/mole.

			TREATMENT PER BEE					
			Dose^a^			Concentration		
Experiment	Feeding schedule	Volume fed (1 M sucrose solution)	Level	µg per feeding	nmoles per feeding	mg/L per feeding	nmoles/L per feeding	mg/Kg (ppm)
*Larval exposure*	Each 24 h for 6 d	2 µl	Control	0	0	0	0	0
	(6 total feedings)	2 µl	Lower	0.033*	0.11*	1.65	57.16	14.64
		2 µl	Higher	0.33*	1.14*	16.5	571.57	146.41
*Adult exposure*	Each 12 h for 3 d	20 µl	Control	0^b^	0	0	0	0
	(6 total feedings)	20 µl	Lower	0.033*^b^	0.11*	1.65*	5.72*	1.46*
		20 µl	Higher	0.33*^b^	1.14	16.5	57.16	14.64

^a^For larvae and adults, each bee was fed a total of 0, 0.2, or 2.0 µg/bee after chronic exposure over multiple days (6 total feedings/bee).

^b^Because adult-treated bees were fed twice each day, daily FLU doses for adults were 0, 0.066*, and 0.66 µg/bee/day.

*Daily doses and concentrations that are field-realistic based upon data collected from foragers (see Methods).

### Larval exposure experiment

#### Flupyradifurone reduced larval survival

**Figure 1:**
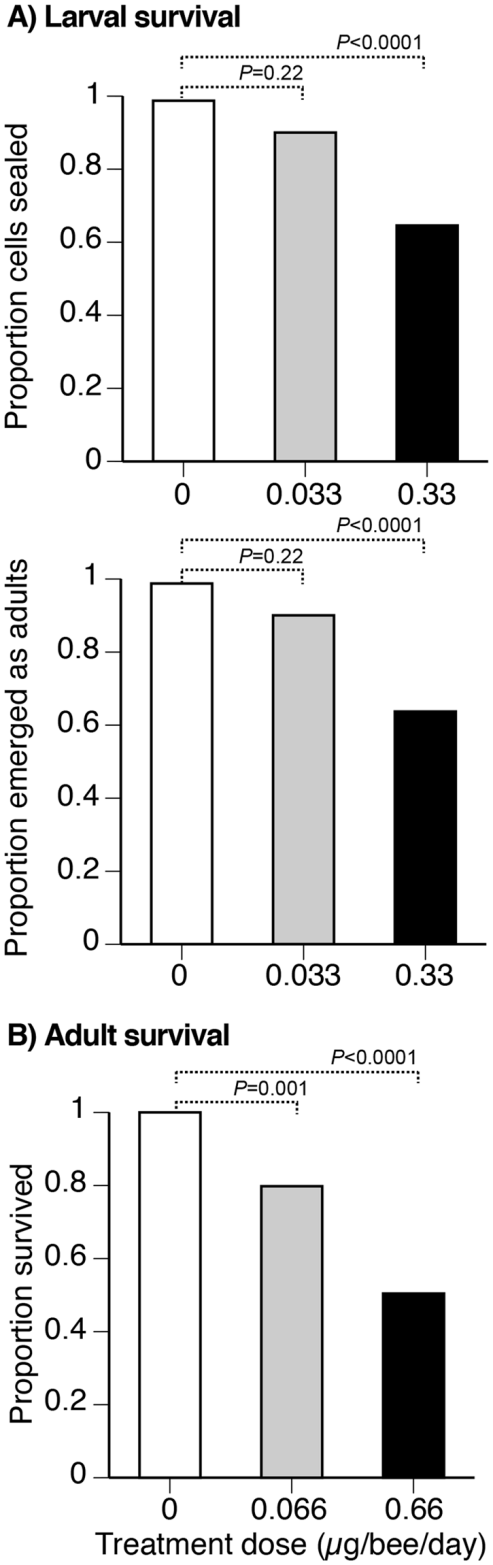
Effects of flupyradifurone on the survival of (**A**) larvae and (**B**) adults. Proportions were calculated based upon the number of initially treated larvae. The *P*-values are from Fisher’s Exact tests, with dashed lines connecting the groups being compared.

We fed larvae for 6 d, beginning at 1 d of larval age. Nearly 100% of control larvae (0 µg FLU) survived to the cell sealing phase and emerged as adults (Fig. 1A). The lower dose of 0.033 µg/bee/day did not significantly alter larval survival to the sealed cell phase or survival to emergence (Fisher’s Exact tests, *P* = 0.22). However, the higher dose (0.33 µg/bee/day) significantly reduced survival to the sealed cell phase (−34%, Fisher’s Exact test, *P* < 0.0001^Dunn-Sidak corrected=DS^) and reduced adult emergence (−35%, Fisher’s Exact test, *P* < 0.0001^DS^) as compared to control bees.

#### Flupyradifurone reduced learning

**Figure 2:**
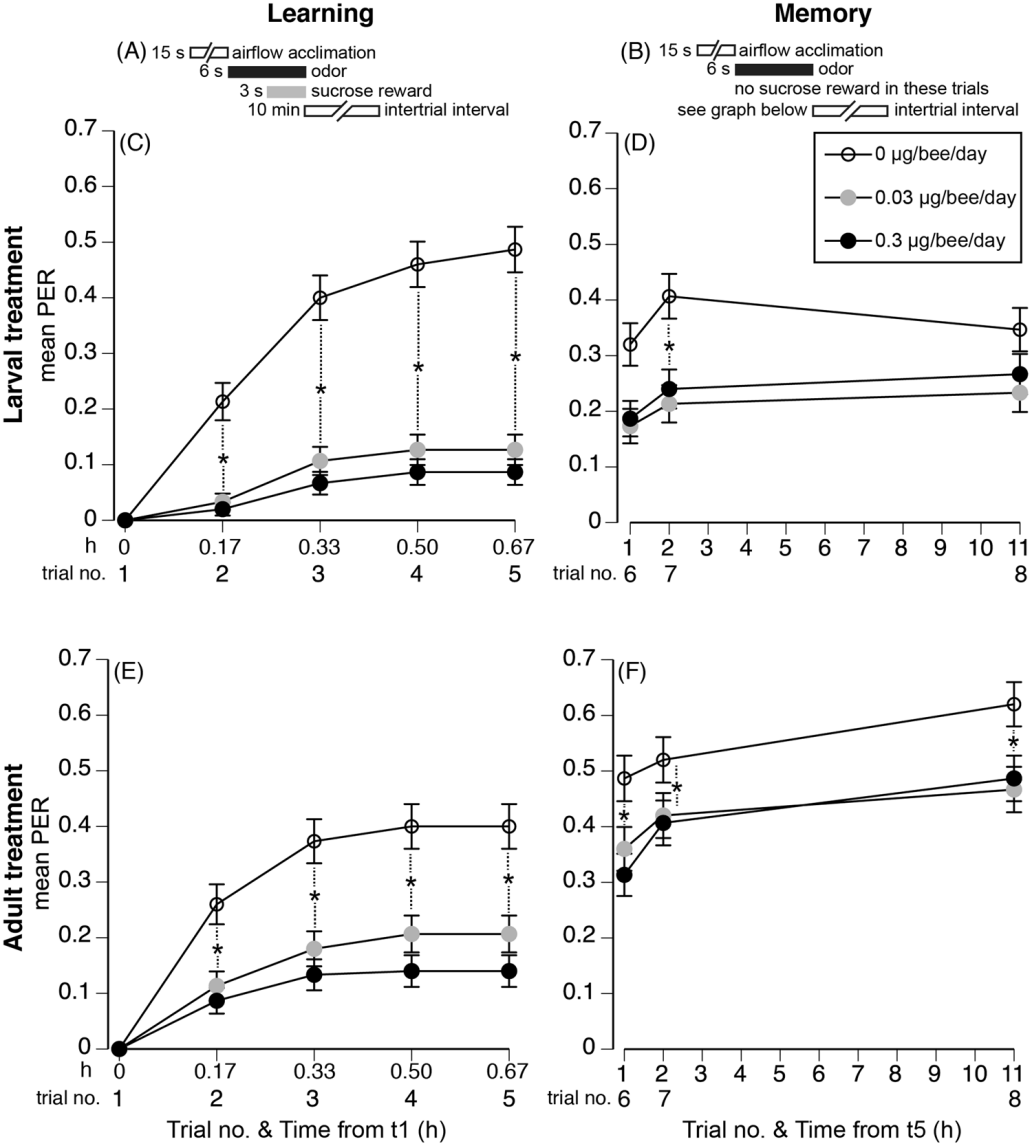
Effect of flupyradifurone (FLU) on olfactory learning and memory (PER) in *A*. *cerana* bees treated as larvae or adults. The temporal design of the (**A**) learning and (**B**) memory trials is shown. For bees treated when they were larvae and tested as adults, we show mean PER for (**C**) learning (elapsed time from first trial shown in h) and (**D**) memory (elapsed time from the last rewarded learning trial, t5, shown). For bees treated and tested as adults (foragers), we also show mean PER for (**E**) learning and (**F**) memory. Dashed lines with stars link points that are significantly different (Tukey HSD tests, *P* < 0.05). Standard error bars are shown. The legend shows the FLU dose that each bee (larva or forager) received per day (see Table 1 for details).

A honey bee exhibits learning when it extends its proboscis (PER) in response to a previously rewarded odor^32^. Larval-treated bees showed learning (significant trial effect: *F*_4,1788_ = 100.14, *P* < 0.0001, Fig. 2C). We then conducted all pairwise comparisons (Tukey HSD test) and report the ones of interest. For each treatment, bees exhibited learning when the 1^st^ trial (before learning) and 5^th^ trial (after four rewarded odor presentations) were compared (Tukey HSD test _t1 vs. t5_, *P* < 0.05). However, control bees (0 µg FLU/bee/day) showed learning after just a single rewarded trial (Tukey HSD test _t1 vs. t2, t3, t4, and t5_, *P* < 0.05). Lower-dose bees (0.033 µg FLU) showed learning only after the 3^rd^ trial (Tukey HSD test _t1 vs. t3, t4, and t5_, *P* < 0.05). Higher-dose bees (0.33 µg FLU/bee/day) learned only after the 4^th^ trial (Tukey HSD test _t1 vs. t4 and t5_, *P* < 0.05). Thus, there was a significant interaction of trial*dose (*F*_8,1788_ = 27.31, *P* < 0.0001) because control bees exhibited higher learning than FLU-treated bees (Fig. 2C). Colony accounted for < 1% of model variance.

Both doses of FLU impaired learning. In each trial that tested learning (t2–5), control bees exhibited significantly higher learning than FLU-treated bees (Tukey HSD test, *P* < 0.05). However, there were no significant differences in learning, per trial, between bees that were fed the lower or higher FLU doses (Tukey HSD test, *P* > 0.05, Fig. 2C).

#### Flupyradifurone treated bees had reduced memory

FLU treated bees were able to show greater average PER responses when their memories were tested (Fig. 2D) as compared to their PER responses during the learning trials (Fig. 2C). However, FLU resulted in lower memory scores as compared to the control (significant dose effect: *F*_2,883_ = 4.99, *P* = 0.007, Fig. 2D). There was a significant overall effect of trial (*F*_2,894_ = 17.93, *P* < 0.0001) because t6 showed lower average PER responses than t7 or t8 (Tukey HSD test, *P* < 0.05). Both lower and higher dose treated bees exhibited lower memory in t7 than control bees (Tukey HSD test, *P* < 0.05). There was no significant interaction of trial*dose (*F*_4,894_ = 0.94, *P* = 0.44). Colony accounted for < 1% of model variance.

### Adult exposure experiment

#### Flupyradifurone reduced adult survival

We captured foragers and fed them treatments over 3 d. Over 3 d of chronic exposure, 100% of control foragers survived. However, only 80% of foragers fed the lower dose of 0.066 µg FLU/bee/day survived as compared to controls (Fisher’s Exact test, *P* = 0.001^DS^). Foragers fed the higher dose of 0.66 µg FLU/bee/day had higher mortality: only 50% survived as compared to controls (Fisher’s Exact test, *P* < 0.0001^DS^). Thus, 0.066 and 0.66 µg FLU/bee/day respectively reduced survival by −20% and −50% after three days of exposure (Fig. 1B).

#### Flupyradifurone reduced learning

Bees treated as adults also showed learning (significant trial effect: *F*_4,1788_ = 109.38, *P* < 0.0001, Fig. 2E). For each treatment, bees exhibited learning when the 1^st^ and 5^th^ trials were compared (Tukey HSD test _t1 vs. t5_, *P* < 0.05). However, control bees (0 µg FLU) showed learning after only a single rewarded trial (Tukey HSD test _t1 vs. t2, t3, t4, and t5_, *P* < 0.05). Lower dose (0.066 µg/bee/day) and higher dose (0.66 µg/bee/day) bees showed learning only after two rewarded trials (Tukey HSD test _t1 vs. t3, t4, and t5_, *P* < 0.05). Thus, there was a significant interaction of trial*dose (*F*_8,1788_ = 10.81, *P* < 0.0001) because control bees exhibited greater learning than FLU-treated bees (Fig. 2E). Colony accounted for <1% of model variance.

Both FLU doses impaired learning. In each trial that tested learning (t2–5), control bees exhibited significantly higher learning than FLU-treated bees (Tukey HSD test, *P* < 0.05). There were no significant differences in learning, per trial, between bees that were fed the lower or higher FLU doses (Tukey HSD test, *P > *0.05, Fig. 2C). Both 0.2 and 2 µg FLU/bee/day were approximately equally detrimental.

#### Flupyradifurone treated bees had reduced memory

Bees fed with FLU showed greater average PER responses when their memories were tested (Fig. 2F) as compared to their PER responses during the learning trials (Fig. 2E). However, FLU resulted in lower memory as compared to the control (significant dose effect: *F*_2,903_ = 5.20, *P* = 0.006, Fig. 2F). There was a significant overall effect of trial (*F*_2,894_ = 5.19, *P* = 0.006) because each successive trial showed higher average PER responses (Tukey HSD test, *P* < 0.05). Both lower- and higher-dose treated bees exhibited lower memory over all trials than controls (Tukey HSD test, *P* < 0.05). There was no significant interaction of trial*dose (*F*_4,894_ = 0.94, *P* = 0.44). Colony accounted for <1% of model variance.

## Discussion

Flupyradifurone (FLU) is thought to have a relatively low impact upon honey bees^24^ and has now been approved for use in multiple countries^21–23^ with a wide variety of crops^19^. However, it’s effects upon bee learning and memory were unknown. We show that sublethal levels of flupyradifurone (FLU) at field-realistic daily doses and concentrations (Table 1) impaired olfactory learning in *A*. *cerana* workers exposed as larvae or as adult foragers. All lower daily doses were field realistic, based upon data collected from foragers (Table 1). The lower dose fed to the larvae (0.033 µg/bee/day) did not alter survival to cell sealing or to adult emergence. However, the lower dose fed to adults (0.066 µg/bee/day), in a separate experiment, significantly reduced survival by 20%. In bees treated as larvae, lower and higher doses of FLU decreased olfactory learning acquisition upon adulthood, on average, by 74% and 82%, respectively (PER at t5). In bees treated as adults, lower and higher doses of FLU decreased olfactory learning, on average, by 48% and 65%, respectively (at t5). FLU-treated bees were able to show somewhat elevated PER responses when their memories were tested and thus the memory impairments, in comparisons with controls, likely arose from reduced learning, not from an impairment of memory alone. However, in bees treated as larvae, lower and higher doses resulted in average memory reductions of 48% and 41%, respectively (t7). In bees treated as adults, lower and higher doses led to average memory reductions of 19% and 22%, respectively (t7). FLU was thus 1.3 to 2.5-fold more harmful to the olfactory learning and memory of bees exposed as larvae as compared to foragers exposed as adults. Further research should therefore be conducted on the effects of FLU, expanding beyond its basic effects on bee survival and colony strength to consider its impact on bee cognition and foraging.

### Field realistic doses and concentrations

Currently available field-realistic data on FLU, a relatively new systemic insecticide, are based upon the exposure of adult foragers, which is reasonable given that foragers may be exposed to FLU as they collect nectar and pollen. The doses that larvae might be exposed to per day are unknown, but are likely a fraction of doses consumed by adults. A worst-case scenario, high field-realistic dose (based upon *A*. *mellifera* collecting nectar from FLU-treated apple blossoms) is 0.44 µg/bee/day^24^. Our lower-dose treatments consisted of 0.033 and 0.066 µg/bee/day given to larvae and adults, respectively, and were therefore only 7.5% and 15% of this high field-realistic dose. Yet both of our low doses resulted in significant learning and memory impairments (Fig. 2). Interestingly, increasing these doses by an order of magnitude to 0.33 and 0.66 µg/bee/day did not result in significantly more impairment. Yang *et al*.^37^ also studied the effects of a nAChR agonist on honey bee learning and reported similar results. They tested olfactory learning of *A*. *mellifera* exposed to imidacloprid as larvae and tested as adults: both 0.04 ng and 0.4 ng reduced olfactory learning by roughly the same degree^37^. Tan *et al*.^43^ similarly studied the effects of imidacloprid on the learning of *A*. *cerana* adults exposed as adults and found that both doses (0.1 ng and 1 ng) similarly reduced learning and memory as compared with controls.

Pesticide exposure durations^47^ and concentrations are important, particularly in experiments in which bees are fed *ad libitum* because bees are being exposed, not to a pre-specified dose, but to a concentration of pesticide that they could gather over multiple days of nectar foraging. Exposure duration is crucial because pesticides can take time in accumulate in an animal’s body^47^. After a single foliar application, FLU residues ≥1.6 ppm have been detected for 3 d in citrus flowers and (after two foliar applications) at ≥3.5 ppm for 3 d in blueberry flowers, up to 110 ppm within 5 d for apple flowers, and 3.5–27 ppm for ≥7 d in apple, melon, and citrus flowers (MRID 48844516^24^). Longer exposure periods are possible. Colonies that have foraged in winter oil seed rape, FLU has been detected for up to five months in the honey and nectar stored in bee combs and for more than two weeks in nectar collected by foragers^24^. Shorter-term exposure is part of long-term exposure to pesticides and is also realistic since bees may not always forage for the entire period of a crop bloom or may not survive, following pesticide exposure, for longer periods. Moreover, lab studies can overestimate pesticide exposure duration^48^. We therefore chose a shorter exposure duration of 3 d for adults, a duration that was already sufficient to elicit a significant effect: 20% mortality at our lowest dose. Our larval exposure duration was based upon the biology of *A*. *ceranae*, whose eggs hatch into larvae and are progressively fed by nurses until their cells are sealed on the 7^th^ day after hatching^49^. We therefore exposed larvae to FLU for 6 d. These conditions also matched a prior study, enabling us to make comparisons with a prior study on the effects of another nAChRs agonist upon *A*. *cerana* olfactory learning^43^.

In terms of FLU concentration, 4.1–4.3 ppm is the worst-case, high end of exposure from nectar consumption, based upon the analysis of forager honey stomach contents^24^. Because we focused on providing specific doses per bee (Table 1), we could only feed larvae a limited volume of sucrose to avoid excessively diluting the nutrients provided in their larval food. We therefore used a higher than field-realistic concentration of FLU (the lower dose corresponded to 14.6 ppm). However, for adult foragers, the lower dose was given at a concentration of 1.46 ppm, which is 35% of the higher field-realistic concentration (Table 1). For larvae, FLU was provided in their brood food, whereas FLU was fed directly to adult mouthparts. It is therefore possible that larvae consumed a smaller amount of FLU than was provided. However, if this occurred, it would demonstrate that FLU fed to larvae has an impact at even lower doses than the ones we tested.

We applied FLU over time and thus the rate at which bee metabolize FLU is also relevant. The metabolism of imidacloprid by bumble bees may provide guidance^50^, if these compounds are similarly degraded. Unfortunately, the details of how honey bees metabolize FLU are not yet known, although an insect cytochrome P450 (CYP6CM1) that confers resistance to neonicotinoids is not able to metabolize FLU^19^. Further studies on how insects degrade and metabolize FLU are needed.

### Effects on larvae vs. adults

In *Apis mellifer*a, olfactory learning ability increases with worker age^51^. We trained bees treated as larvae at 7 d of adult age and foragers at foraging age (approximately 20–22 d)^49^. However, our observed learning differences likely did not arise because of these age differences. In fact, control larval-treated bees (50% PER _t5_) showed somewhat higher learning than control adult-treated bees (40% PER _t5_, Fig. 2). Tan *et al*.^43^ found a similar level of learning in control bees treated as larvae (32% PER _t5_), but greater learning in control bees treated as adults (72% PER _t5_). Memory showed similar trends in our study and in Tan *et al*.^43^. Multiple factors, including genotype^52^ and seasonality^53^, influence honey bee PER learning. Our differences in control bee learning may therefore have arisen because we used different colonies and studied our bees over a different range of months than Tan *et al*.^43^.

We designed our experiments to feed larvae and adults the same total amounts of FLU over multiple days of exposure. Per day, larvae were fed half the dose given to adults because prior research suggested that larval honey bees are more sensitive to pesticides than adults^37,43^. However, despite receiving a smaller daily dose (and the same total dose per treatment), larvae showed more severe memory impairment (Fig. 2). These results suggest that larvae are more susceptible to FLU than adults. Likewise, Tan *et al*.^43^ showed that imidacloprid, another nicotinic acetylcholine receptor agonist, had a stronger effect when consumed by larvae as compared to adult *A*. *cerana*. These effects extend to other species. *Apis mellifera* larvae fed quite low doses of imidacloprid had significantly impaired olfactory learning^37^, comparable to impairments that required much higher doses fed to adults^46^. Our results suggest that we should continue to explore effects of pesticide exposure during bee development.

### Future directions

A recent study reported that *A*. *cerana* colonies reared in agricultural areas with intensive use of multiple pesticides had an impaired ability to detect odors^54^. Our experiments suggest that FLU impaired olfactory learning, not olfaction *per se*, because adults fed FLU were able to recover, to some degree, memory when tested with the rewarded odor (Fig. 2E,F). However, further studies are required to determine if olfaction is also impaired. Determining the potential colony-level effects of FLU on *A*. *cerana* are also important, given that *A*. *cerana* may be more susceptible than *A*. *mellifera* to pesticides that act on nicotinic acetylcholine receptors^27,28^. Our results suggest that such studies would be worthwhile, particularly if they consider the potential effects of larval exposure and how individual cognitive impairments may manifest at a colony level.

## Methods

We conducted two experiments and used three different colonies per experiment, for a total of six *A*. *cerana cerana* colonies at Yunnan Agricultural University, Kunming, China from September 2015 to March of 2016. To facilitate comparisons with a prior study in *A*. *cerana*, both experiments are closely based upon Tan *et al*.^43^, which tested the effect of the neonicotinoid pesticide, imidacloprid, chronically fed to larvae or adults upon olfactory learning.

### Flupyradifurone doses and concentrations

Glaberman and White^24^ summarize the typical FLU concentrations and doses that bees encounter when foraging on pesticide-treated crops. The LD_50_ of FLU (the dose at which half of exposed bees die over a specified time period) is 1.2 µg/bee for pure FLU and 3.4 µg/bee for the seed treatment formulation^24^. Some of the highest concentrations (0.44 µg/bee/day) are reported in apples given a foliar spray^24^. Field-realistic high FLU concentrations of 4.3 ppm and 4.1 ppm were found in the honey stomachs of foragers collecting nectar from oilseed rape treated with FLU at manufacturer-recommended application levels MRID 48844516 and MRID 48844517 in ^24^.

In our experiments, we chronically fed bees fixed doses of analytical grade flupyradifurone (Sigma Aldrich, CAS# 951659–40–8, catalog# 37050-100MG) to ensure that each bee received the same dose (Table 1). Each week, we prepared fresh solutions with double-distilled water from a concentrated stock solution. All solutions were stored at 4° C and wrapped in aluminum foil to prevent potential light degradation.

Our goal was to achieve the following total FLU doses administered over multiple days: 0 µg/bee (control), 0.2 µg/bee (lower), and 2.0 µg/bee (higher) in 1 M sucrose solution. We used 1 M sucrose solution (30% sucrose w/w) to provide a better comparison with a prior *A*. *cerana* study^43^ and because honey bees typically collect floral nectar in the range of 19–60% (w/v)^55,56^. Larvae were fed over 6 d and therefore received 0, 0.033, or 0.33 µg FLU/bee/day. Adults were fed over 3 d and received 0, 0.066, or 0.66 µg FLU/bee day. Larvae were fed a lower range of doses because prior research showed that honey bee larvae may be more sensitive to nicotinic acetylcholine agonists than adult bees^37^. Bees were fed FLU at concentrations of 0, 1.46, 14.6, or 146.4 ppm, depending upon the experiment (Table 1).

### Effects of larval exposure to FLU upon subsequent adult learning and memory

To determine the effects of FLU fed to larvae, we used three different colonies. We took 50 bees per colony per treatment (150 bees/treatment). We used three treatments and tested a total of 450 bees.

To treat the larvae, we removed a brood comb (one comb per treatment) from each colony, overlaid it with a clear acetate sheet, and marked where the queen had laid eggs. We then returned this comb to the colony. After the eggs had hatched (3 days later), we removed the comb and gently pipetted 2 µl of treatment solution into the larval food of each cell (which now contained a 1-day old larva). Each larva was fed a 1 M sucrose solution containing 0, 0.033, or 0.33 µg FLU/bee/day for 6 days (0 ppb, 14.6 ppb, or, 146.4 ppb, Table 1). This resulted in total doses of 0, 0.2, or 2 µg/bee over the 6 days of chronic exposure. We chose 2 µl because preliminary trials showed that larvae readily consumed this entire volume. We simultaneously tested all three treatments with each colony.

In each colony, for each treatment dose, we fed approximately 250 larvae to obtain a sufficiently large sample size to examine the mortality effects of FLU (total of 2334 larvae treated). However, we only tested PER learning in a randomly selected subset of these bees.

On the 7^th^ day, when *A*. *cerana* cells are normally sealed^43^, we removed control and pesticide-treated combs, counted how many of the treated cells were sealed, and removed all of the larvae and pre-pupae that were not in any treatment group to ensure that only treated bees emerged. Each comb was placed in a different box in the same incubator (35 °C at 60% humidity) for about 10 days until adult bees emerged. We counted the number of bees that emerged from each treatment.

These newly emerged workers were maintained in the incubator with the food naturally stored in their respective combs until they were 7 days of adult age. A random subset of bees was then removed, each placed in an individual vial, cold anesthetized on ice for 1–2 min until their movements substantially diminished, and then placed in a modified plastic centrifuge tubes with just the heads exposed for PER testing method of ^44^.

We then placed each bee in the olfactory conditioning apparatus and familiarized it with the primary, unscented airflow (50 mL/s) for 15 s^43,57^. Bees were exposed to the olfactory conditioned stimulus (CS) via a secondary air flow (2.5 mL/s) through a Pasteur pipette into which we inserted a clear filter paper strip with 10 µL of pure hexanal (Sigma-Aldrich, 98% pure, CAS# 66-25-1, Lot# MKBG1555V).

We used a modified olfactory conditioning protocol^43^ based upon Bitterman *et al*.^58^. We presented the CS only for 3 s (scoring proboscis extension, PER, during this time), and then presented the unconditioned stimulus (US = 1 M pure sucrose solution containing no pesticide) for 3 s. In the rewarded learning trials, the CS continued such that CS and US overlapped for 3 s (Fig. 2A). To assess learning, we conducted five rewarded learning trials (t1–5), each with a 10 min intertrial interval. To assess memory, we subsequently tested responses to 3 s of odor alone with no sucrose reward (Fig. 2B) at intervals of 1 h (t6), 2 h (t7), and 11 h (t8) after the last rewarded learning trial (t5)^43^. Less than 5% of bees had a spontaneous PER response to odor only in t1 (before learning could have occurred) or showed no PER after antennal stimulation with 1.0 M sucrose. We excluded these bees from our analyses (standard protocol of ^58^.)

### Effects of adult exposure to FLU upon adult learning and memory

For this experiment, we used a different set of three colonies and only fed FLU to adult foragers, not larvae. As in the larval experiment, we also tested 50 bees per colony per treatment (150 bees/treatment, a total of 450 bees for the three treatments). We captured likely foragers by slowly and carefully approaching a colony to avoid alarming guard bees and using a clean plastic bottle to capture foragers as they flew away from the nest entrance^43^.

We cold-anesthetized these foragers and restrained them as described above. Restrained adult foragers were fed 20 µl of sucrose solution containing one of the three treatments, all in 1 M sucrose. Each adult bee was fed twice a day (each 12 h) over 3 d to achieve 0, 0.06, or 0.66 µg FLU/bee/day and total doses of 0, 0.2, and 2.0 µg FLU/bee after 3 d of exposure (Table 1). During these 3 d, they were incubated at 35 °C at 60% humidity. Bees were also incubated under the same conditions for 1 h after their last feeding to allow pesticide absorption. We ran all three treatment groups in parallel during each trial. As in the larval experiment, < 5% of these bees were excluded^58^ because they showed spontaneous PER to odor before conditioning or failed to show PER upon antennal stimulation.

### Statistics

We used JMP v12 Pro statistical software. For each experiment, we separately analyzed learning acquisition (t1–5) and memory (t6–8, Fig. 2). We used repeated-measures, REML algorithm Analysis of Variance (ANOVA) to analyze the fixed effects of treatment (nominal variable) and trial (ordinal variable). Colony and bee identity were included in each model as random effects. To explore the detailed effects of treatment and trial, we used Tukey Honestly Significant Difference (HSD) tests, which correct for potential Type I statistical error^59^. To determine if the pesticide-treated bees had lower rates of survival than control bees, we used Fisher’s Exact 2-tailed tests^60^ calculated using GraphPad online software (https://graphpad.com/quickcalcs/contingency1.cfm). For these tests, we applied the Dunn-Sidak correction^59^ because each pesticide-treated group was compared with the same control group (*k* = 2). We designated tests that passed the Dunn-Sidak correction as ^DS^.

### Data availability

All data are available in the supplemental data spreadsheet.

## Electronic supplementary material

Supplementary Dataset
